# The Effectiveness of Manual Therapy in the Cervical Spine and Diaphragm, in Combination with Breathing Re-Education Exercises, on the Range of Motion and Forward Head Posture in Patients with Non-Specific Chronic Neck Pain: A Randomized Controlled Trial

**DOI:** 10.3390/healthcare13141765

**Published:** 2025-07-21

**Authors:** Petros I. Tatsios, Eirini Grammatopoulou, Zacharias Dimitriadis, George A. Koumantakis

**Affiliations:** 1 Physiotherapy Department, School of Health & Care Sciences, University of West Attica (UNIWA), 12243 Athens, Greece; igrammat@uniwa.gr; 2 Laboratory of Advanced Physiotherapy, Physiotherapy Department, School of Health & Care Sciences, University of West Attica (UNIWA), 12243 Athens, Greece; 3 Health Assessment & Quality of Life Laboratory, Physiotherapy Department, University of Thessaly, 35100 Lamia, Greece; zdimitriadis@uth.gr

**Keywords:** musculoskeletal, pain, biomechanics, mHealth, craniovertebral angle, neck mobility, respiratory

## Abstract

**Background/Objectives:** A randomized controlled trial (RCT) was designed to test the emerging role of respiratory mechanics as part of physiotherapy in patients with non-specific chronic neck pain (NSCNP). **Methods:** Ninety patients with NSCNP and symptom duration >3 months were randomly allocated to three intervention groups of equal size, receiving either cervical spine (according to the Mulligan Concept) and diaphragm manual therapy plus breathing reeducation exercises (experimental group—EG1), cervical spine manual therapy plus sham diaphragmatic manual techniques (EG2), or conventional physiotherapy (control group—CG). The treatment period lasted one month (10 sessions) for all groups. The effect on the cervical spine range of motion (CS-ROM) and on the craniovertebral angle (CVA) was examined. Outcomes were collected before treatment (0/12), after treatment (1/12), and three months after the end of treatment (4/12). The main analysis comprised a two-way mixed ANOVA with a repeated measures factor (time) and a between-groups factor (group). Post hoc tests assessed the source of significant interactions detected. The significance level was set at *p* = 0.05. **Results:** No significant between-group baseline differences were identified. Increases in CS-ROM and in CVA were registered mainly post-treatment, with improvements maintained at follow-up for CS-ROM. EG1 significantly improved over CG in all movement directions except for flexion and over EG2 for extension only, at 1/12 and 4/12. All groups improved by the same amount for CVA. **Conclusions:** EG1, which included diaphragm manual therapy and breathing re-education exercises, registered the largest overall improvement over CG (except for flexion and CVA), and for extension over EG2. The interaction between respiratory mechanics and neck mobility may provide new therapeutic and assessment insights of patients with NSCNP.

## 1. Introduction

Non-specific chronic neck pain (NSCNP) constitutes a substantial and intricate musculoskeletal disorder in contemporary society, ranking as the fourth leading cause of disability and exhibiting an annual prevalence surpassing 30% [1]. Given the multifactorial nature of neck pain, a range of risk factors can contribute to its onset [2]. Furthermore, the peak in point prevalence observed in many studies occurred during middle age [3,4].

Patients with chronic neck pain exhibit altered neuromuscular control due to diminished strength of deep neck flexor and extensor muscles combined with hyperactivity and increased fatigability of superficial neck flexors and certain pain characteristics (nociceptive and cognitive–psychological), which may contribute to respiratory dysfunction [5,6]. The aforementioned pain-related dysfunctions contribute to the perpetuation of forward head posture (FHP), reduce active range of motion (ROM), and lead to inefficient or altered breathing patterns, often associated with hypertonia and shortening of accessory respiratory muscles, as well as a documented reduction in the mobility of the thoracic cage [7,8].

The primary objectives of physiotherapy treatment are to alleviate pain, minimize disability, restore ROM and FHP [9], and enhance motor control [10]. Physiotherapists routinely perform comprehensive physical examinations to evaluate functional limitations and effectively track the progress of rehabilitation interventions. Guidelines established by the American Physical Therapy Association (APTA) underscore the necessity of employing reliable measurement tools to assess changes in a patient’s functional status throughout the course of neck pain treatment [11]. The cervical spine range of motion (CS-ROM) is one such measure specifically recommended by the APTA [11,12]. The assessment of CS-ROM is a standard clinical method used to evaluate and classify individuals with neck pain [13], identify any functional limitations, and provide valuable prognostic information [14]. Research examining predictors of recovery in chronic neck pain has suggested that higher levels of anxiety and limited range of motion during lateral neck flexion may indicate a greater probability of positive results from manual therapy based on the Mulligan concept [15]. FHP describes a deviation where the head is positioned more anteriorly than its ideal alignment, which would typically be along a theoretical vertical plumb line extending perpendicularly from a horizontal line passing through the body’s center of gravity, and is considered a postural deviation imposing increased mechanical stress on the tissues surrounding the cervical spine [9].

While a substantial proportion of individuals with NSCNP seek physiotherapy interventions, a single, universally effective treatment for neck pain does not exist [16]. These interventions often include traditional physiotherapy, patient education, general exercise programs, or manual therapy techniques (such as diaphragmatic or spinal mobilization, manipulation, and specific exercises), and respiratory exercises [10]. However, a considerable number of these patients experience a stagnation in their recovery and do not achieve complete resolution of their symptoms [17].

In recent years, the potential of manual therapy and breathing exercises as a therapeutic tool for managing pain and musculoskeletal dysfunctions has garnered increasing attention [10,18,19]. Manual therapy exerts its effects on improving both musculoskeletal and respiratory outcomes through the activation of various biomechanical, neurophysiological, psychological, and other non-specific mechanisms in patients with NSCNP [20]. Research into the positive impacts of breathing exercises on pain management indicates their ability to modulate pain perception and enhance body alignment [20]. Specifically, diaphragmatic breathing is believed to lessen the sensation of pain by reducing activity in the sympathetic nervous system and to alleviate muscular tension, consequently leading to improved posture and contributing to overall well-being [18,21]. Furthermore, evidence suggests that breathing exercises may help lower pro-inflammatory markers, highlighting their potential in reducing inflammation [22]. A recently conducted systematic review found no randomized controlled trials that combined cervical and diaphragmatic manual therapy with breathing re-education exercises as a treatment approach in patients with NSCNP [7].

The aim of this study was to evaluate the effects of a combination of cervical spine and diaphragm manual therapy plus breathing re-education exercises compared to cervical spine manual therapy plus sham diaphragmatic manual techniques versus conventional physiotherapy on active CS-ROM and CVA.

## 2. Materials and Methods

### 2.1. Sample

A non-probability sample of ninety participants, ranging in age from 25 to 65 years, were recruited on a voluntary basis for this study, which took place between June 2022 and August 2023. The sample size was determined using the G*Power program version 3.1.9.7 for an a priori power analysis [23], conducted for a repeated measures ANOVA (within–between interaction) with a medium effect size f = 0.25, a power of 0.95, and an alpha (α) error level of 0.05. The minimum sample size calculated by G*Power amounted to 54 participants. It was increased further to allow for a 10% drop out rate, as well as by taking into account the central limit theorem, inferring that at least 30 participants per group would be required for the sample to be considered to have characteristics of a normal distribution [24]. Participants’ inclusion and exclusion criteria are presented in detail in a previous publication [25].

### 2.2. Ethics

Ethical approval for this study was obtained by the Ethics Committee of the University of West Attica, Greece (51758—1 June 2022), which was conducted according to the Declaration of Helsinki stipulations. The trial was registered with ClinicalTrials.gov under the identifier NCT05229393 (https://clinicaltrials.gov/study/NCT05229393, accessed on 1 May 2022).

### 2.3. Study Design

This study was a randomized controlled trial (RCT) with a blinded assessor (single-blinded), designed to test the hypothesis that the combination of breathing exercises plus cervical spine and diaphragm manual therapy would be more effective than cervical spine manual therapy or conventional physiotherapy in patients with NSCNP.

### 2.4. Procedures

All participants completed a written questionnaire on demographic information and their symptoms’ duration and underwent a standardized assessment, including a detailed medical history and a battery of specialized orthopedic tests (Spurling’s test, traction test, upper limb tension test, and shoulder abduction test). One rater (A.T.), with more than 20 years’ experience as a physical therapist, performed all measurements, following a standardized measurement protocol. Outcomes were collected before and after the treatment period lasting one month (10 treatment sessions), and 3 months post-treatment.

Following all initial assessments, participants were randomly allocated to one of the three intervention groups via a computer-generated random number sequence block randomization method with specialized software (http://www.randomizer.org/ (accessed on 10 May 2022)), using blocks of three participants. Participants were assigned covertly, using sealed, sequentially numbered opaque envelopes [26]. An independent trial manager not involved in the recruitment, assessment, or intervention delivery, performed the randomization and concealed allocation processes. The researcher in charge of carrying out the treatment programs only opened each participant’s envelope at the time of their intervention initiation.

### 2.5. Interventions

A comprehensive account of all interventions administered in this study is available in the study’s protocol [10]. Their content is briefly outlined below.

#### 2.5.1. The Cervical Spine and Diaphragm Manual Therapy Plus Breathing Exercises Group (Experimental Group 1—EG1)

Cervical spine manual therapy was administered, consisting of inter-vertebral mobilization techniques of the cervical spine (Natural Apophyseal Glides—NAGs, Sustained Natural Apophyseal Glides—SNAGs, and traction techniques, according to the Mulligan Concept) [27], along with soft tissue techniques (stretching of cervicothoracic muscles); then, diaphragmatic manual therapy was applied (doming diaphragmatic technique and diaphragmatic release technique [28], followed by breathing re-education exercises [29].

#### 2.5.2. The Cervical Spine Plus Sham Diaphragmatic Manual Therapy (Experimental Group 2—EG2)

Cervical spine manual therapy was administered, mirroring the protocol used for EG1. They also underwent sham diaphragmatic manual therapy, with a therapeutic ultrasound device (Sonopuls 692 Enraf-Nonius) while seated, with the device activated, but its intensity set at 0 W/cm^2^, giving the impression of treatment without any actual therapeutic effect, for 10 min [30,31]. The sham ultrasound procedures were consistently performed by the same therapist at the area of the diaphragm. Additionally, oral instructions on integrating postural corrections into routine daily activities, such as cleaning and sweeping, were provided.

#### 2.5.3. Conventional Physiotherapy Group (Control Group-CG)

Participants in CG were administered a standard physiotherapy protocol. The intervention commenced with Transcutaneous Electrical Nerve Stimulation (TENS), applied for 15 min to the suboccipital region and the upper-middle trapezius muscles bilaterally, using a pulse duration of 250 microseconds and a frequency of 80 Hz. Subsequently, pulsed microwave diathermy was administered via an Enraf-Nonius RADARMED 950+ device for 10 min, with the participant in a seated position. The session concluded with 15 min of deep soft tissue massage, employing slow-speed gliding and kneading techniques. This massage therapy specifically targeted the upper, middle, and lower fibers of the trapezius, along with the splenius capitis and levator scapulae muscles.

#### 2.5.4. Adverse Events Assessment

A specific form was constructed to record any potential adverse effects of therapy during and after the physiotherapy treatment, according to a 5-level rating [32], considering the type of adverse event (fatigue, muscle discomfort, stiffness, pain increase, nausea, headache, dizziness, or other), its intensity, and its duration. Should any patient have presented with serious adverse events, they would have been withdrawn from the trial and referred back to their doctor for further evaluation.

### 2.6. Outcomes

#### 2.6.1. Cervical Spine Range of Motion

A detailed description of active CS-ROM measurement in all movement directions with the KFORCE SENS^®^ KINVENT (Montpellier, France) electrogoniometer as followed in the present study, and its test–retest reliability evaluation, is provided in a preceding study [25].

#### 2.6.2. Forward Head Posture

The Craniovertebral Angle (CVA) is a frequently used method for evaluating FHP [9]. A CVA angle of less than 50 degrees represents more severe FHP [33]. A detailed description of the methodology employed in the present study for its assessment and its test–retest reliability, as a measure of forward head posture, is provided in a previous study [25].

### 2.7. Statistical Analysis

All outcomes were analyzed per group for normality of distribution using the Shapiro–Wilk Test. Baseline between-group comparisons were made (one-way ANOVA and chi-square tests) to establish whether the applied randomization procedure was successful. The main analysis was performed with a 3 × 3 two-way mixed ANOVA [24] with one repeated measures factor (time), and a between-groups factor (group) was used to analyze each of the outcomes separately. Mauchly’s test was used to assess the sphericity assumption. Post hoc tests were employed to determine the source of any significant interactions detected, specifically, univariate tests to examine within-group differences between time-points and between-group differences at each of the time-points. The Bonferroni correction method was applied to adjust *p* vales for the multiple comparisons post hoc tests employed, reducing the possibility of type I error. The significance level was set at *p* = 0.05. Partial eta squared (η^2^p) effect sizes were used to determine the percentage of variation in the data that could be attributed to treatment differences. The power achieved per outcome was also reported.

All analyses were performed according to the ’intention-to-treat’ (ITT) principle, with all subjects randomly assigned for intervention analyzed in their assigned groups. Missing data for ITT analyses were handled by inserting group means in the place of missing values, a relatively conservative approach [34]. Data were analyzed using the IBM SPSS 29.0.2.0.

## 3. Results

### 3.1. Demographics

Overall, 90 patients (54 women) with NSCNP with a mean (SD) age of 41.25 (10.93) years, who were referred for physiotherapy at a private practice, participated in this study. Each patient had a current pain episode that lasted longer than three months, with a mean (SD) of 6.39 (1.72) months. Participants’ detailed demographic characteristics per group allocation are displayed in Table 1. 

No statistically significant differences were identified between groups in either their demographic (Table 1) or their clinical baseline characteristics (Table 2). Only three participants (all from the control group) dropped out after having completed all treatments, two without providing a reason and one because of moving abroad.

### 3.2. Outcomes

In general, all outcomes were normally distributed in 55 out of the 63 analyses performed (7 outcomes × 3 groups × 3 measurement occasions). Examination of the Q-Q plots for the eight sets of variables that the Shapiro–Wilk test was significant revealed minimal deviations of the standardized values along their regression line; therefore, the outcomes were considered acceptable for parametric analysis.

**Figure 1:**
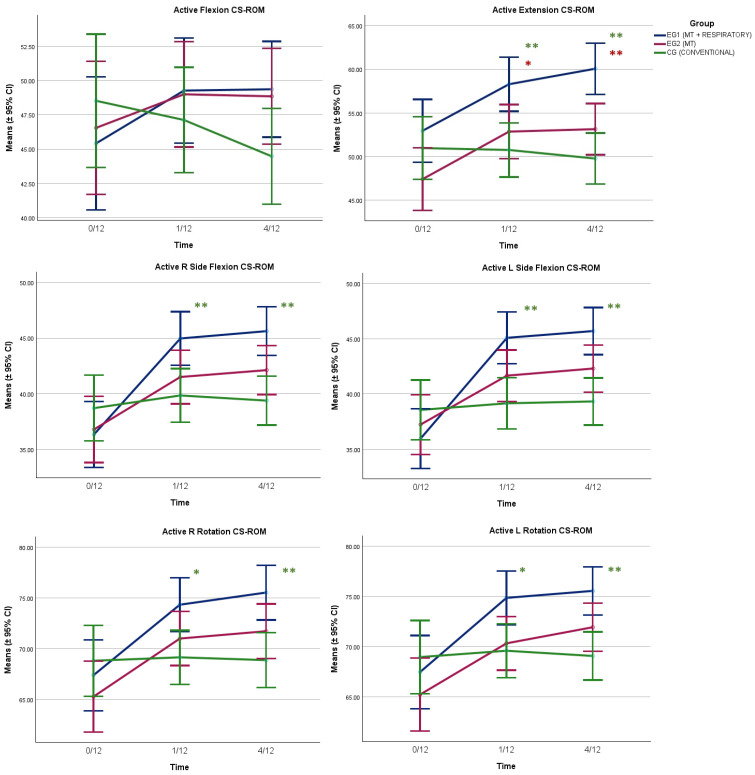
Change over time of active CS-ROM per movement direction for the three groups of participants. The univariate analysis (one-way ANOVA) applied at each time-point, with post hoc pairwise comparisons (*p* values adjusted with the Bonferroni correction method), identified significant differences between EG1 and EG2 (red asterisks) and EG1 and CG (green asterisks) where noted. * *p* < 0.05, ** *p* < 0.01.

**Figure 2:**
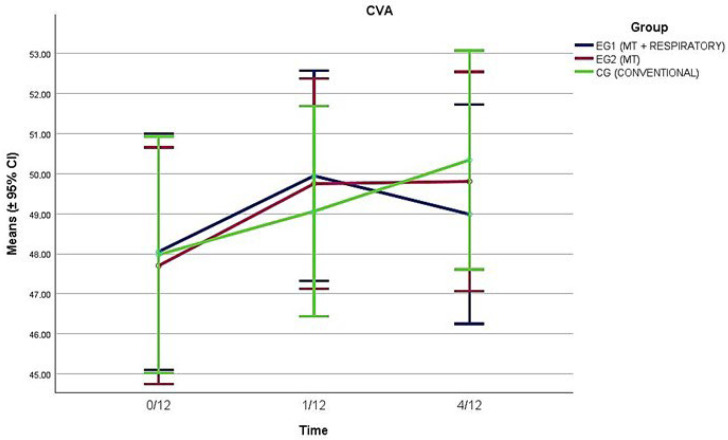
Change over time of the craniovertebral (CVA) angle for the three groups of participants.

All CS-ROM outcomes improved with time (*p* < 0.001), with the main improvements identified for EG1 and EG2 between baseline and 1/12 and maintained at 4/12 (Table 2 and Figure 1). Significant interaction effects (time × group) were registered for all CS-ROM directions (*p* < 0.001), with EG1 registering significant improvements over CG, and over EG2 for extension CS-ROM (Figure 1). CVA improved with time also (*p* < 0.008); however, there were no interaction effects registered (*p* = 0.50), with all groups improving by the same amount (Figure 2).

Post hoc pairwise comparisons (one-way ANOVA) applied at each time-point, with *p* values adjusted with the Bonferroni correction method, specifically identified significant differences between EG1 and EG2 only for extension CS-ROM (time-point 1/12: ΔM (95% CI) = 5.43° (0.03°, 10.83°), *p* = 0.048, and time-point 4/12: ΔM (95% CI) = 6.93° (1.83°, 12.03°), *p* = 0.004) (Figure 1). In addition, significant differences were recorded between EG1 and CG for five out of six movement directions (the exception being flexion), and specifically for extension (time-point 1/12: ΔM (95% CI) = 7.54° (2.14°, 12.94°), *p* = 0.003, and time-point 4/12: ΔM (95% CI) = 10.34° (5.21°, 15.40°), *p* < 0.001); for R side flexion (time-point 1/12: ΔM (95% CI) = 5.13° (0.93°, 9.33°), *p* = 0.01, and time-point 4/12: ΔM = 6.26° (2.43°, 10.08°), *p* < 0.001); for L side flexion (time-point 1/12: ΔM = 5.94° (1.88°, 9.99°), *p* = 0.002, and time-point 4/12: ΔM (95% CI) = 6.38° (2.67°, 10.09°), *p* < 0.001); for R rotation (time-point 1/12: ΔM (95% CI) = 5.19° (0.56°, 9.83°), *p* = 0.02, and time-point 4/12: ΔM = 6.67° (1.98°, 11.35°), *p* = 0.002); and for L rotation (time-point 1/12: ΔM (95% CI) = 5.27° (0.63°, 9.92°), *p* = 0.02, and time-point 4/12: ΔM = 6.49° (2.33°, 10.65°), *p* < 0.001) (Figure 1).

## 4. Discussion

The results of this study show that a combination of cervical spine and diaphragm manual therapy plus breathing re-education exercises (EG1) led to superior improvement of active CS-ROM compared to conventional physiotherapy (CG) for five out of the six movement directions examined. There was also a trend for superior improvement in EG1 versus EG2 (cervical spine manual therapy plus sham diaphragmatic manual techniques) in those movement directions (except for flexion); however, between-group comparisons revealed statistically significant differences only for extension CS-ROM. The improvements noted for EG1 and EG2 in CS-ROM were maintained 3 months post-treatment. Conversely, there were no between-group differences in the improvement in either EG1 or EG2 over CG in CVA, as all groups noted a similar small range of improvement, of around 2° on average, over the study period.

Patients with NSCNP can present with altered neck muscle activation and poor coordination, contributing to FHP [9], limited active neck motion, and altered breathing patterns [5,35,36]. FHP can lead to pain and elevate the likelihood of degenerative changes and disc herniation [36]. Neck pain linked to FHP is often associated with poor postural habits, occupational demands, and structural impairments [37]. Furthermore, the resulting changes in breathing can trigger reduced tissue oxygenation, increased neuromuscular excitability, and accessory muscle changes (hypertonia, shortening, thoracic hypomobility) [38]. This sequence establishes a vicious cycle that feeds into the experience of NSCNP pain [9,10].

Therefore, a combined approach may be more effective in patients with NSCNP. Firstly, a manual therapy approach such as the one based in the Mulligan Concept, applying NAGs and SNAGs in the zygapophyseal joints that guide spinal movement, is believed to increase the range of motion (ROM) [39]. The rationale behind these techniques when initially proposed was considered to be primarily biomechanical, since, by correcting ‘positional faults’ through repositioning the superior articular facet with SNAGs, clinicians aim to reduce pain and enhance the ROM. Restoring normal joint mechanics may subsequently, therefore, normalize adverse neuromotor coordination responses caused by restricted joint movement [15]. However, findings from a current living review of systematic, narrative, and scoping reviews indicate that applying manual therapy stimulates a range of complex, multisystem responses. These responses include not only biomechanical, neurovascular, neurological, neurotransmitter/neuropeptide, neuroimmune, neuroendocrine, and neuromuscular mechanisms but also others [40]. Two RCTs involving patients with NSCNP have tested the effectiveness of Mulligan manual therapy on CSROM and CVA [41,42]. In both studies, the Mulligan manual therapy group demonstrated statistically significant improvements in all CS-ROM movement directions examined, and the CVA.

Furthermore, manual therapy targeting the diaphragm has not only demonstrated immediate improvements in diaphragmatic movement but also, through its relationship with the phrenic nerve, exhibits a hypoalgesic effect on the cervical spine [43]. Another potential mechanism of interaction between the diaphragm and the cervical spine may be via a fascial link, specifically via the transversalis fascia [44] and the thoracolumbar fascia [44,45]. In support of this fascial interconnection, the reviewed literature frequently highlights an immediate improvement in posterior muscle chain flexibility as a key outcome following diaphragmatic manual therapy [46]. However, consistent with our findings, a randomized clinical trial performed in healthy adults indicated that a single session of diaphragm stretching significantly enhanced cervical extension, right and left cervical flexion, posterior chain flexibility, and xiphoid-level ribcage excursion, outperforming a placebo technique that utilized disconnected ultrasound [30].

According to a recent systematic review [19], slow breathing significantly influences autonomic regulation, cardiovascular function, and overall physiological well-being. Across various studies, these methods consistently improved measures like heart rate variability, respiratory sinus arrhythmia, baroreflex sensitivity, and even cortical synchronization. This reinforces their potential to therapeutically boost vagal tone and fine-tune autonomic balance. Physiologically, these benefits stem from the baroreflex mechanism and the activation of vagal afferents via pulmonary stretch receptors during extended exhalation. This process enhances respiratory sinus arrhythmia and adjusts brain–heart interactions through the nucleus tractus solitarius.

In parallel, diaphragm manual therapy and breathing re-education exercises focus on releasing the dome of the diaphragm to enhance its descent, increase the mobility of the rib cage, and reduce tension in the accessory inspiratory muscles (scalenes, sternocleidomastoid, subclavius, intercostal muscles, and levatores costarum) [43,47]. Consequently, these effects combined should contribute to improved head posture and cervical spine mobility.

Our systematic review, conducted prior to the initiation of this clinical study [7], identified four randomized trials [48,49,50,51] that investigated the effectiveness of breathing exercises focusing on CSROM [48,49,51] and on CVA [50] in patients with NSCNP. In all those trials evaluating CSROM, statistically significant improvements were observed in specific movements, such as flexion and extension, but not consistently across all movements. Also, in line with our trial, regarding CVA, the study by Dareh-Deh et al. 2022 showed considerable improvement in all three groups, but no statistically significant difference was observed between the experimental groups and the control group [50]. Finally, another RCT that assessed the effectiveness of breathing exercises in individuals with Chronic Neck Pain (CNP) [52] registered a statistically significant between-group difference in active neck extension and FHP immediately post-treatment and at two weeks post-treatment, following a set of 10 treatment sessions over two weeks. The above findings suggest that perhaps respiratory exercises alone are not sufficient to enhance CVA or CS-ROM consistently in all directions of movement.

Although our results are in agreement with most studies, we identified heterogeneity regarding the type of respiratory exercises applied (pursed lips breathing, relaxation exercises according to Jacobson, diaphragmatic breathing exercises, VODIS, and balloon breathing exercises), the duration of treatment (ranging from 2 to 8 weeks), the number of sessions per week (1–5 weeks), the total number of sessions (3–40) and the duration of patients’ chronicity, which no study explicitly reported.

This is the first study that has examined the combined effect of cervical spine manual therapy with diaphragm manual therapy and breathing re-education exercises on active CS-ROM and CVA in patients with NSCNP. Regarding the quality of the measurements of this study, a recent systematic review with meta-analysis [53] mentioned that smartphone applications like clinometer, compass, and other, have proven to be reliable tools for measuring CS-ROM in both people with and without neck pain, offering physiotherapists a valuable resource. However, as the overall quality of the research supporting these applications was identified as ‘limited’ [53], we have verified the within-day test–retest reliability and construct validity of both CS-ROM and CVA in half of the study participants [25].

Among the limitations of our study is the absence of a longer-term follow-up period, specifically extending to one year, to assess the sustained effects of this treatment approach. Investigating the long-term impact of such an intervention on patients with NSCNP would provide valuable insights. Also, the perceived credibility of the deactivated ultrasound applied as sham intervention in EG2 may have differed in perceived credibility compared to hands-on manual therapy; however, the therapist behavior and setting were standardized to enhance the credibility of this intervention. For ITT analysis, missing values were imputed using group means, a method selected for its simplicity, transparency, and ability to preserve sample size without excluding participants from analysis. While we acknowledge that this approach may underestimate variability and is less robust than modern techniques such as multiple imputation, it is considered acceptable in cases of minimal amounts of missing values. This conservative method was deemed appropriate for our dataset, in which the proportion of missing data was low (3.33%), and sensitivity analyses using complete cases showed comparable results.

The selection of CS-ROM and CVA as primary outcomes was guided by the biomechanical focus of the present manuscript, which aimed to evaluate objective changes in cervical posture and mobility. Therefore, clinical outcomes such as pain intensity, disability, quality of life, and psychological outcomes were not included. Furthermore, other biomechanical and neuromotor control variables relevant to neck pain that were not measured, such as neck muscle strength, endurance, proprioception, and thoracic spine range of motion might have provided a more comprehensive clinical picture of this patient group, and potentially may have offered further insights into the relationships between different musculoskeletal-based variables. Finally, the fact that we did not progressively implement any respiratory muscle strengthening exercises possibly constitutes another limitation of the current study and should also be investigated. Future studies are recommended to clarify these potential mechanisms and to determine the duration of the observed effects, preferably by examining a larger population and including follow-up assessments. This may assist in identifying knowledge gaps and guide future research endeavors.

## 5. Conclusions

This study represents the first single blind randomized controlled trial to demonstrate and quantify the extent of improvement in CS-ROM and in CVA, after applying a combined intervention of cervical spine manual therapy with diaphragm manual therapy and breathing re-education exercises in patients NSCNP. Furthermore, the combination of diaphragm manual therapy plus breathing re-education exercises constitutes an easily applicable, more holistic, low-cost, and effective intervention that can provide additional effectiveness regarding CS-ROM in patients with NSCNP who have reached a therapeutic plateau with conventional manual therapy and exercise regimens. This research further supports the notion that interventions applied to distant somatic tissues, such as the diaphragm, can positively influence a relatively remote painful area, such as the cervical spine, through their common innervation, fascial structural continuity, and improvement in autonomic regulation, vagal tone, and stress resilience.

## Figures and Tables

**Table 1:** Descriptive statistics (mean ± SD) of participants’ demographic characteristics per treatment group.

	EG1 (*n* = 30)	EG2 (*n* = 30)	CG (*n* = 30)	Statistic	*p*
Sex (Male/Female)	15/15	8/22	13/17	x^2^ = 3.61	0.16
Age (y)	41.20 ± 11.53	39.93 ± 8.00	42.63 ± 12.87	F = 0.45	0.64
Height (m)	171.93 ± 7.53	171.20 ± 6.24	172.00 ± 9.85	F = 0.09	0.91
Body Mass (kg)	74.50 ± 18.01	73.97 ± 14.94	77.30 ± 13.99	F = 0.39	0.68
Body Mass Index (kg/m^2^)	24.96 ± 4.63	25.11 ± 4.07	26.10 ± 4.18	F = 0.62	0.54
Pain duration (months)	6.47 ± 1.57	6.43 ± 1.89	6.27 ± 1.72	F = 0.11	0.89

y: years, kg: kilograms, m: meters.

**Table 2:** Descriptive statistics (mean ± SD) and interaction effect (time × group) of six CS-ROM movement directions, and CVA for all measurement time-points per treatment group.

Outcomes	EG1 (n = 30)	EG2 (n = 30)	CG (n = 30)	F (4, 172)	*p*	η^2^	Power
CS-ROM							
Flexion (^ο^)							
Pre (0/12)	45.42 ± 12.74	46.55 ± 14.82	48.52 ± 12.53	8.78	<0.001	0.17	0.99
Post (1/12)	49.27 ± 10.30	48.99 ± 11.38	47.12 ± 10.03				
Follow-up (4/12)	49.36 ± 9.37	48.85 ± 10.32	44.47 ± 9.13				
Extension (^ο^)							
Pre (0/12)	52.96 ± 10.48	47.42 ± 9.45	50.97 ± 9.86	8.03	<0.001	0.16	0.99
Post (1/12)	58.29 ± 7.91	52.86 ± 9.13	50.75 ± 8.62				
Follow-up (4/12)	60.07 ± 7.72	53.14 ± 8.85	49.77 ± 7.63				
R Side Flexion (^ο^)							
Pre (0/12)	36.32 ± 7.51	36.77 ± 6.63	38.69 ± 9.99	7.43	<0.001	0.15	0.99
Post (1/12)	44.96 ± 6.82	41.50 ± 6.18	39.83 ± 6.96				
Follow-up (4/12)	45.63 ± 5.84	42.11 ± 5.42	39.37 ± 6.85				
L Side Flexion (^ο^)							
Pre (0/12)	35.94 ± 7.90	37.22 ± 6.17	38.54 ± 8.13	7.01	<0.001	0.14	0.99
Post (1/12)	45.08 ± 6.87	41.65 ± 5.45	39.14 ± 6.88				
Follow-up (4/12)	45.70 ± 5.90	42.30 ± 5.18	39.31 ± 6.50				
R Rotation (^ο^)							
Pre (0/12)	67.38 ± 10.17	65.27 ± 6.60	68.81 ± 11.51	6.04	<0.001	0.12	0.98
Post (1/12)	74.34 ± 6.15	71.00 ± 6.36	69.15 ± 9.16				
Follow-up (4/12)	75.54 ± 6.00	71.73 ± 6.07	68.87 ± 9.64				
L Rotation (^ο^)							
Pre (0/12)	67.45 ± 10.75	65.21 ± 8.16	68.95 ± 10.99	5.63	<0.001	0.12	0.98
Post (1/12)	74.85 ± 6.41	70.32 ± 6.68	69.58 ± 8.78				
Follow-up (4/12)	75.55 ± 5.99	71.93 ± 5.49	69.06 ± 8.05				
CVA (^ο^)							
Pre (0/12)	48.05 ± 6.90	47.70 ± 8.35	47.98 ± 9.04	0.84	0.50	0.02	0.26
Post (1/12)	49.95 ± 6.73	49.75 ± 7.21	49.06 ± 7.75				
Follow-up (4/12)	48.99 ± 6.29	49.81 ± 8.10	50.34 ± 8.08				

CS-ROM: cervical spine range of motion, CVA: craniovertebral angle, R: right, L: left, °: degrees.

## Data Availability

The data presented in this study are available upon request from the corresponding author. The data are not publicly available due to the applicable data protection law in Greece (Law 4624/2019).

## Abbreviations

The following abbreviations are used in this manuscript:

BMI	Body Mass Index
CG	Control Group
CS-ROM	Cervical Spine Range of Motion
CVA	Craniovertebral Angle
EG	Experimental Group
FHP	Forward Head Posture
L	Left
NSCNP	Non-Specific Chronic Neck Pain
R	Right
